# Taking the learning beyond the individual: how reflection informs change in practice

**DOI:** 10.5116/ijme.52ec.d21f

**Published:** 2014-02-08

**Authors:** Fiona Muir, Mairi Scott, Kevin McConville, Kenneth Watson, Kazem Behbehani, Faten Sukkar

**Affiliations:** 1 uTayside Centre for General Practice, School of Medicine, University of Dundee, UK; 2 Student, School of Medicine, University of Dundee, UK; 3 Dasman Diabetes Institute, Kuwait; 4 Department of Education and Training, Dasman Diabetes Institute, Kuwait

**Keywords:** Health professional education, multi-professional, reflection, international

## Abstract

**Objectives:**

The purpose of this research was to explore the value of reflection and its application to practice through the implementation of educational modules within a new Diabetes Care and Education Master Degree Programme in Kuwait, and to realise how this teaching intervention informs changes in practice.

**Methods:**

A small exploratory case study was conducted within the Dasman Diabetes Institute, Kuwait. A qualitative approach using focus group interviews was carried out with seventeen participants all of whom are studying on the Diabetes Care and Education Master Degree Programme in Kuwait. An inductive approach to thematic analysis, which focused on examining themes within data, was performed.

**Results:**

The results indicate that participants value the opportunity to study through organised, structured and assessed reflection. The learning provides useful information and support to the participant by highlighting the role which reflection plays to enhance personal and professional development, the value of educational theory, continuing professional development, collaboration and enhancing patient education and practice.

**Conclusions:**

The significance of reflection is often seen in the literature as an important aspect of professional competence. This research has highlighted the value of reflection as a key component within a new educational programme.

## Introduction

There are significant and increasing number of patients who suffer from diabetes mellitus in Middle East Gulf States and this along with therapeutic advances in diabetes care, requires practitioners to continually enhance their knowledge and skills. Consequently a new approach to the organization and delivery of diabetes education was needed. The Dasman Diabetes Institute invited The University of Dundee and NHS Tayside to join in partnership with them to create a new educational program grounded in excellent, modern clinical care, designed to meet the needs of healthcare and laboratory practitioners in Kuwait. The programme started recruiting students in September 2011. Approximately one hundred participants are enrolled each year. Working through the programme at Masters Level the participants extend their subject knowledge and strengthen their analytical, problem-solving and communication skills thereby increasing their professional effectiveness. Learning is applied through work-based study and project activity which are relevant to healthcare and laboratory practitioners working in Kuwait, in both public and private sectors. By achieving a qualification in Diabetes Care and Education, graduates are well placed to contribute to the prevention, management and understanding of this serious and widespread disease in Kuwait and in addition to contribute to a transformational change to Kuwait’s health care delivery system.Participants on the programme choose a personal study pathway from four key modular areas: clinical provision, education, research and management. A variety of modules exist within these categories to allow students to choose the most appropriate topics for their stage of study and learning needs. Modules are units of study of twelve weeks duration.Students progressing to MSc must successfully complete a core Diabetes Care module (or Science of Diabetes module for non-clinically based practitioners), four specialist modules covering a range of educational, organizational and clinical topics, and a Research Methods module.The mandatory on-line core introductory module consists of essential preparatory work which covers baseline knowledge, learning methods and study protocols along with additional reading that can be completed during their first few months on the programme. The module aims to be a common basis in order to establish the concepts of learning styles, information skills, academic writing skills, as well as to introduce problem-based learning and reflection.Reflection is an important aspect of the course having been introduced during an induction conference, ‘The Discovery Conference’ using the concept of a ‘Reflection tree’ and additionally throughout all modules by encouraging the participants to consider how their practice compares to the themes explored in the modules.^1^ The importance of reflection is frequently noted in the literature as an essential characteristic for professional competence.^2^^-^^5^ Reflective learning has the intention of improving learning and when this happens, in the context of working with the ill-defined problems of professional practice, it is often called reflective practice. Reflective practice is a form of practice which seeks to reappraise many situations of professional performance so that practitioners can continue to learn, grow and develop in and through practice.^6^ With respect to reflection in education and a growing requirement from professional bodies to enhance the development of professional competence, it is important to recognise the ways in which theory and practice are provided and linked.^7^^,^^8^Education is considered to be a process of development and growth, the aim of which is to develop the individual to the utmost of their potential.^7^ Dewey maintained that sound educational experience involves continuity and interaction between the learner and what is learned.^7^ Education must be considered as a continuing reconstruction of experience; that the process and the goal of education are one and the same thing. Thus, the continuity of experience or what may be called the experiential continuum is to discriminate between those experiences which are worthwhile educationally and those which are not.Thus, the ‘principles of reflective writing’ module informs the student about reflection, a concept that underpins each module within the programme. All modules have two summative assessed assignments. Assignment one, a 2000 word report relates to a specific workplace based project based on the module subject topic which is conducted over six weeks. Assignment two of every module assessment is a 1500 word reflective analysis of the participant’s professional learning as they undertake the project work for each module. The Post Graduate Certificate/Diploma modules have a clear common module framework including key theories and content through preparatory reading, face-to-face teaching and online study activities; students develop projects addressing some of the key theories/content in relation to their individual professional healthcare setting. In this context, knowledge is constructed through the experience and cumulative acquisition, selection and interpretation of the experience.^8^ Students who complete the Postgraduate Diploma Diabetes Care and Education can commence the MSc Taught Dissertation module.Whilst several writers have looked at interventions using reflective learning techniques and strategies to develop the participant’s reflective skills and abilities to evaluate new and existing programmes, there appears to be limited research into the value of reflective learning within the practitioner’s context in Kuwait.^3^^,^^9^^-^^11^Whilst previous research has considered reflection in teacher training programmes in United Arab Emirates there appears to be little research pertaining to its value within the postgraduate healthcare and laboratory practitioners’ context (Kuwait).^10^This study is different in character to other studies as it explores the value of reflection within a new educational programme from a Kuwait perspective. The aim of the present study was to:

- Explore the participant’s experiences of reflection in terms of teaching and learning.
- Explore the significance of reflection for practice including personal and professional development.
- Identify what, if anything, the participant would like to see changed in the educational delivery method.

## Methods

### Design

An exploratory case study approach was adopted to reflect the interactive nature of the experiences of participants in one educational setting.^11^ Three focus group interviews with participants involved in the programme were conducted. One of the distinct features of focus-group interviews is its group dynamic. Hence the type and range of data generated through the social interaction of the group are often deeper and richer than those obtained from one-to-one interviews.^12^ Group interactions between participants gave insight into attitudes, opinions and perceptions.^13^^,^^14^

### Participants

The participants in this study were healthcare and laboratory practitioners on the programme who had something to say on the topic, and were comfortable talking to the interviewer and each other. The total number of participants was fourteen, male (three) and female (eleven). The participants included medical staff, laboratory practitioners, nurse supervisors, general practitioners, and a nutritionist. Their professional experience ranged from two to twenty-three years.The programme is intended to enable those working in the field of diabetes to gain sound evidence-based knowledge of the clinical, education and organisational components of modern diabetes care, thus enabling them to effectively implement a high quality evidence-based clinical service. Students have a good knowledge of English although it is not their first language. Many have studied internationally and are familiar with western universities.The education is delivered by a range of professionals: medical consultants, diabetes nurse specialists, general practitioners, and educationalists. Three focus groups were carried out: group one n=eight, group two n=six, group three n=three, group. The recommended number of people per group is usually six to ten although some researchers have used as few as four.^14^^,^^15^ Numbers of groups vary, some studies using only one meeting with each of several focus groups, others meeting the same group several times.^16^ Each group was interviewed once and each interview lasted approximately sixty minutes. Data was collected until data saturation occurred. Saturation of data was evident when a range of ideas were heard, recurrent patterns and themes emerged and no new information gleaned.Participants were recruited through the participant year lists 2011-2013. Data was derived from the one case and not selected on a random basis. Kvale advocates that one conducts interviews with as many people as necessary to gain the information required.^17^ The reason for choosing these individuals was that they were easily accessible. We are directly involved in the teaching of participants.

### Procedure

Ethical considerations applied throughout the research. Ethical approval at the university level (University of Dundee) and local research ethics committee was obtained before the research began.^18^Confidentiality and anonymity assurances were given so that the rights of the individuals were not compromised. Participants were given an introductory letter via email with the details of the study and its purpose and the format of the focus group interview. They were given the opportunity to decline participation in the study before arrangements were made for attending the focus group meeting. Queries were answered verbally and written consent was obtained. This provided a degree of proof that the participant was aware of the nature of the study and they had given informed consent.We ensured that full information was given about the purpose and use of participants’ contributions. Being honest and keeping participants informed about the expectations of the group and topic, and not pressurising participants to speak was deemed good practice. A particular ethical issue considered in the focus groups was the handling of sensitive material and confidentiality as there was more than one participant in the group. At the outset the facilitator clarified that each participant’s contribution would be shared with the others in the group and the facilitator. Participants were encouraged to keep confidential what they heard during the meeting and we assured anonymity of the data from the group.A pilot study was conducted with staff who would not be participating in the main study. The focus group research involved organised discussion with a selected group of individuals to gain information about their views and experiences of the topic. Thus, focus group interviewing was particularly suited for obtaining several perspectives about the same topic. The benefits of the focus group research include gaining insights into people’s shared understandings of everyday life and the ways in which individuals are influenced by others in a group situation.An interview guide was used to provide a framework for the interview and maintain consistency of the questions asked as it is in the comparison and contrast that themes and patterns emerge. In analysis, we strived for theoretical saturation which is only possible with consistency of questioning.^19^Participants were given a brief introduction to the purpose of the study and an explanation of what they were being asked to do. The participants were interviewed in a place convenient to them and for as long as required. This took on average approximately sixty minutes for each group.

### Analysis

Focus group analysis is a deliberate, purposeful process in which it is systematic, uses verifiable procedures, is done in a sequence and is an on-going process.^19^ Interviews were recorded, transcribed verbatim then listened to again along with the transcriptions for accuracy. The data was then analysed using an analytical process which involves a number of interconnected stages to classify and organise the data according to the key themes, concepts and emergent categories.^20^ This data was supplemented by field notes. To ensure internal validity reflexivity, triangulation, peer judgment and validation of the data with the interviewees were considered.^21^ Reflexivity is the continuous process of reflection by the researcher on anything in the research environment: his own values, pre-conceptions, behaviour or presence and those of the participants, which may affect responses. It is a self-awareness of the relationship between the investigator and the research environment.^22^ Thus, we considered their own interactions with the participants, the way in which the participants were prompted and probed during questioning and the sequence of the questions; reappraised coding of the responses, interpreting the recording of the data/transcripts and the handling of the interviews. We are members of the teaching staff within the programme therefore we were aware that our presence could have affected the situation. Participants may have wanted to impress, avert or control the situation. However, this effect was reduced by ensuring as much as possible a careful presentation of the self.Denzin suggests triangulation involves using more than one method to gather data, such as interviews, observations, questionnaires, and documents to increase confidence in the interpretation.^23^ Thus, narrative analysis of the students’ reflective coursework was carried out in conjunction with focus group data analysis.To further confirm the validity of data interpretation a peer debriefing method was implemented whereby data was secondarily analysed by an impartial source. Resulting analyses were compared to ensure concurring themes, and decrease the likelihood of researcher bias.

## Results

Due to the number of responses the results from the three participant groups have been combined. The main themes identified in the analysis are as follows: educational theory, continuing professional development, patient care/ education and practice, collaboration and support, value of modules, self-awareness /personal development, developing critical awareness.

### Educational theory

Participants were aware of a process for reflecting. They used reflective strategies, such as models or frameworks, to guide them through the process. Participants acknowledged the various tools available to them, as suggested by their programme tutors, and via the educational learning zone which is accessible to all students. An awareness of educational theory and preparation were regarded as necessary to meet the module requirements. Within the programme the student’s reflective ability is assessed by written evidence of their experiences.

“…Learning Zone …Jenny Moon model as a … structured model” Student 2: Group 2

“Kolb…Gibbs …its experimental… I use it during my course…the framework from a [participant] guide and then we add some guides” *Student 6: Group 2*

### Continuing Professional Development

The prospect of education was deemed as an ‘opportunity for lifelong learning’, ‘to engage in further study’; to gain new experiences while learning to improve their knowledge and skill, ‘an opportunity to ‘drive change.’

“… it has to do with the value that I place on education and the commitment that I have made for lifelong learning…” *Student 4: Group 1*

“I enjoy continuing education…so it's part of my habit...the only difference was with Dundee is that reflection is part of it” *Student 3: Group 3*

### Enhancing patient care/education and practice

Participants related to the importance of reflection for enhancing patient care and how, through reflection, practice can be changed to benefit both the patient and practitioner. Participants identified the difficulty they had experienced with managing change for both patient care and procedures in the workplace. This new found learning had provided the skill and knowing ‘how to make a difference’.

“…from the feedback from the patient[s] themselves, or the staff themselves, they came out with a good knowledge, or they learn something, or the patient can manage properly his diabetes, even if he knew very simple information. And mostly we will feel really so happy that we did some changes” *Student 5: Group 1*

“If some patient refuse some line of medication, we can take it step-by-step as [an] educationalist and also through reflection [explore] why he is refusing it… with the reflective practice … I can manage it much better… a benefit for the patient and me [as a practitioner]” *Student 3: Group 3*

### Collaboration and support

Participants related to the way in which reflection had generated a culture of collaboration with colleagues and the importance of communication to inform change for future practice. Participants had either not considered working with others or found this aspect a challenge in their workplace. Thus interactive learning, by way of encouraging adult learners with active engagement in learning, was evident.

“…Improvement of the lab staff, their knowledge, their skills…to educate the community about diabetes and the good things like social habits that we have to change, and to learn about the risk. …improvement for, like, small community in our work and then our work will come to the large community in Kuwait as well” *Student 3: Group 1*

“…develop the communication between primary care sections and the Dasman…we put the evidence in front of all of us and everyone is starting in neutral to evaluate this event then everyone is brave enough to say his opinions and we can collect the information and reach a consensus, rather than telling them, ‘what happened today is wrong, it should not happen again’. It is better to say, ‘why this happened, how can we improve this in the future’… and everyone will share in the decision” *Student 2: Group 2*

### Significance of the modules

The merit of undertaking the study modules to enhance their facilitative/teaching role to inform best practice; develop new knowledge and skills; integrate theory to practice was identified by the majority of participants. Participants agreed the ways in which this ‘new’ learning, through reflection, had influenced their approach to teaching.

“Where I saw a big difference with this was when we were doing the 'Training the Trainer' [module]…as a health educator and as a health promoter… I applied the different theories of adult learning – andragogy, pedagogy… reflection…and you really put thought into what you were giving to the people and how the people would be receiving it, you had…much more impact in your delivery” *Student 4: Group 1*

“I came as a diabetes nurse educator but … I never make PowerPoint… so I learn with my colleague, or all of us we learn, the best practice through reflection… the main things… I really got from the 'Training the Trainer'… role play and… the positive reflection, because we are as Arab and I'm sorry to say [we don’t do] that [reflection]. We…start with negative…so I learn how to start with positive. So it was really, really, very, very important thing for me to add this new information or these new skills” *Student 5: Group 1*

### Self -awareness and personal development

The majority of participants linked the importance of reflection to their self-development; ‘an awareness of yourself’; increased confidence; communication style; personal and professional life and highlighting it as ‘a habit’, ‘a part of life’. It was viewed as a significant milestone for personal and professional development.

“…having done this for five courses – my fifth course now –..the reflection aspect of the assignment has led me to personally and professionally grow, to really put more thought into what it is that I do and what it is that I say” *Student 4: Group 1*

“…we used to sit all together, all the nurses… most of us were nurses… we used to sit together with a doctor… other speciality like public relation, lab… and everybody in Dasman…. Somebody would check my way or our way of talking. And we used to take it very… we didn't take it, like, personally. We just take it that we want to improve ourselves” *Student 5: Group 1*

### Developing critical awareness

Developing a critical awareness through an ability to reflect enhanced the participants questioning of themselves, and practice, to find new meaning. This enabled a deeper understanding of a situation to extract the implications for future practice.

“It makes you think…as physicians we are calculative and we have numbers, and also it does help us to imagine a bit and think out of the box, and analyse it in another way, not by numbers… by feelings and expressions. So it makes you imagine a bit” *Student 4: Group 2*

“… besides 'I', I use 'why' in my reflection. I write 'why' … You must explain everything. .. You must go in deeper …” *Student 6: Group 2*

## Discussion

The findings of this study show the value of understanding reflection, its application and relevance to future practice for continuing personal and professional development, lifelong learning and patient care/education.The role of reflection, in the on-going professional development of participants to support practice was transparent. It would seem that the education, by focussing on the development needs of the participant and interactive learning, supports reflective skill proficiency.Distinct from other studies this case study findings illuminated ‘reflection’ for diabetes education in Kuwait. Learning, as indicated by individuals, through formal activities provided on-going preparation and support to reflect on practice. Participants accepted that there was an expectation from the programme tutors that they could and would reflect as part of the programme. This expectation incorporated formalised learning in that the reflective learning was organised, structured and assessed. Participants acknowledged that the process for reflecting, as part of the programme assessment, was transparent whilst the importance of reflection to their profession helped defined their learning needs.Participants of this Master degree programme are required to be self-directed learners, demonstrate better thinking skills, and critically appraise of evidence which is informed by ‘modern educational theory.^24^^,^^25^ This was evident by the way in which participants questioned established practice and so created depth to their learning.^26^ Being a critical thinker is a key component of personal development and fostering critical thinking is crucial to creating and maintaining a healthy social equality.^27^ Critical thinking implies being aware of the assumptions central to our actions and responses, paying attention to the context within which our actions and ideas originate, questioning single answers to problems and being open to alternative ways of looking at and acting in the world.^28^Of particular significance was the identification that participants knew of reflective strategies which can be used to structure the reflective process and developed their knowledge and skill of reflecting. They had received formal training and education in reflection which enhanced their ability to critique and analyse a situation, and learn from it. Participants in this study receive guidance from the outset of their programme of study. This endorses the view of other writers who suggest that there is a need for reflection to be incorporated early in the programme and to value reflective practice as a core professional skill.^24^^,^^26^^,^^29^The challenge for educationalists will be to accumulate a repertoire of strategies which they can implement with different people.The Gulf Cooperation Council CPD Alliance has highlighted that practitioners need to know of the benefits of reflection and the need to be critically reflective as part of on-going professional development and lifelong learning.^29^ Thus, developing new meanings from educational experiences through selection, organisation and interpretation should enhance the quality of such experiences and take practitioners to a higher level of cognitive and affective learning that enhance their competence.^30^Reflection on work based practices enabled the acquisition of new knowledge by reviewing and learning from experience, arising from action and problem solving, within a working environment. The learning is centred round live projects and challenges to individuals and organisations. The creation of knowledge, as a shared and collective activity, is one in which people discuss ideas and share problems and solutions.^31^Developing confidence, enhancing communication and receiving feedback from tutors and peers, as key features, fit with previous evidence. Roche and Coote acknowledged that reflection improved confidence and clinical reasoning whilst self-awareness, and hence self- improvement, requires feedback from reliable sources.^32^^,^^33^ In this study collaborating with others to benefit the community, by exchanging views and opinions and learning from each other was meaningful. In support of this argument, Boud recommends that reflection should not be an isolated activity.^34^This programme has encouraged practitioners in Kuwait to consider the theoretical knowledge which is required to support their practice. The qualitative research described has identified relevant issues and concepts in terms of the value of reflection and reflective guidance to develop the participant’s reflective ability through a core module and on-going reflective assessments. This finding is supported by Williams who concludes in her study of radiographers within a new postgraduate programme, that all participants valued a core module on reflective practice Learning from reflective thinking is not automatic; it requires active involvement and a clinical environment that is supportive towards the learner’s needs.^35^^,^^36^The small sample size of participants, in exploring the value of reflection and its application to practice, is a limitation of the study. The study aimed to explore and gain an appreciation of the case in its setting, its totality and its complexity. Thus, this rich description should enable readers to see whether the study is applicable or not to their situation which may in turn be a foundation for future research.

## Conclusion

Compared with previous work, this methodological approach added value as it facilitated a rich exploration of participants’ experience of reflection in the ‘here’ and ‘now ’within the Kuwait context. The successful implementation of reflective learning and rewarding yield for practitioners, with implications for patient care, is important from an educational and clinical perspective.The next step is to utilise the findings from this study to develop and enhance the current programme. Future research also is needed to investigate the longer term value and implications for both staff and patients.

## 

### Conflict of Interest

The authors declare that they have no conflict of interest.
